# The Liver, Viewed as a Sugar-Producer

**Published:** 1855-04

**Authors:** William Howell


ART. III. — *The Liver, viewed as a Sugar-producer. A Thesis, presented to the Faculty of the University of Buffalo.* By WILLIAM HOWELL. Published by order of the Curators.

The following observations are based principally upon notes taken of Dr. John C. Dalton's lectures on Physiology, during the winter of 1853–4. Claiming no originality, and desiring only to present a *resumé* of the present state of physiological knowledge of the subject treated of, advantage has also been taken of the various reports of M. Bernard's lectures, by Donaldson, Atlee, Parkes and others, and of his works, as published by himself. Coming freshly, as did Dr. Dalton, from the teachings of M. Bernard, to whose researches and additions nearly every department of physiology owes so much, his lectures, vivified as they were by the enthusiasm of a true lover of science, possessed peculiar interest. And particularly were they interesting to the young student, who, as yet, had experienced none of the necessary everyday realities of the practice of his profession: realities so eminently destructive of the romantic and fine-drawn theoretical. A realm was opened to him so wondrously new, and so variedly interesting, that it was little wonder that the beautiful New should ravish his attention from the venerable Old, stern in its venerability. Yet as this New could not fail to be received by the mind to which it was presented, almost by intuition, as true, the time devoted to its culture could not be illy spent.

Among the wonderful additions which M. Bernard has made to all the branches which he has cultivated, to none other has he been of so great service as to the function of Digestion. From its paramount importance he has made it his particular study, and to him, more than to any other observer, do we owe the present advanced state of science in regard to it. Indeed, before his labors, almost no experimental observations had been made, if we except the crude efforts of Spallanzani, the better directed researches of Magendie, and those of Beaumont, who nobly commenced what Bernard has carried so far on toward its completion. Bernard, however, by the establishment, at will, of fistulous openings into the stomach, and into the ducts of all the secretory and excretory organs, possessed a signal advantage over Beaumont, who was obliged to restrict himself to the observation of the action of the stomach and its secretions. He was thus enabled to prove, that though most of Dr. Beaumont's ideas in regard to the function of Digestion were true, some of them, (such as the gastric juice being the sole solvent, &c.,) were not entitled to so much consideration. As one after another of the results of his researches were given to the world, with each new gift to science, admiration for the student, and respect for his work, increased. But in 1848, the announcement of the brightest of his physiological achievements astonished the scientific world, and it is difficult to decide, which most to admire, the zeal with which he pursued his experiments, or the sagacity which he displayed in interpreting from their results the great fact that sugar, so generally found in the vegetable kingdom, exists also in the animal, as a constant secretion, and not as derived from saccharine or amylaceous ingesta. As vegetables do not find it already formed in the earth, but eliminate it for themselves, so, by experiment, he proved its production by the normal and habitual physiological action of the liver.

Pathological phenomena, as manifested in the disease, diabetes mellitus, first drew his attention to the subject. It was well known, that besides starch ($C^{12}$ $H^{10}$ $O^{10}$) there were three kinds of sugar, taken into the economy, in the aliment, the grape-sugar, or glucose ($C^{12}$ $H^{14}$ $O^{14}$) the cane-sugar ($C^{12}$ $H^{11}$ $O^{11}$) and the milk-sugar ($C^{12}$ $H^{12}$ $O^{12}$). That of these the only assimilable form was the glucose, to which the others are readily convertible, by the addition to, or deprivation of, certain equivalents of water (H O) in the stomach. In the case of the cane-sugar, which may be absorbed as cane-sugar, there appears to be a special provision in the liver for its conversion, after being absorbed by endosmose, into the form (namely, glucose) in which its presence in the blood can be best tolerated.

It had always been supposed that when sugar was found in the blood or any of the secretions, it must have been derived from food which contained it, or from which it could be formed. It was considered an established fact, that the protein or azotized compounds could be placed under neither of these heads. The idea obtained with physiologists that the animal organism was incapable of creating, *de novo*, any principle found within it, but only possessed the power of destroying that which was furnished by the vegetable world. Hence they asserted the impossibility of sugar being manufactured by the organism, which was only capable of destroying that which came from without. Upon this idea was based that part of the treatment of diabetes, which consisted in withholding from the patient all amylaceous articles of diet. But though this method sometimes cured, and always ameliorated the disease, it seemed to Bernard a remarkable fact, that while the most absolute restriction to protein ingesta was maintained, the urine of the patient should continually remain heavily loaded with sugar. To ascertain whence this sugar was derived, and what was the mode of its formation, he instituted a series of experiments, and, after two years of the most laborious investigation, he conclusively demonstrated, that in a very large proportion of animals, the liver is constantly engaged in the formation of sugar, from either the azotized or non-nitrogenous materials furnished to it by the portal blood. That this production is continued at all periods, and independently of digestion, though its quantity is increased during the action of that function.* That it takes place at the expense of the blood, which is incessantly traversing the liver, and that it is prevented by the destruction of the nerves supplying that organ. That when the liver does not act, as in severe fevers, sugar is not formed, showing that the liver must be in a physiological condition of action. That in abstinence the production of sugar gradually diminishes until death, and after no more sugar is found, when the liver is no longer acting, although the animal takes food, it is too late, he certainly dies.

Some idea may be formed, as to the extent of the labor performed by M. Bernard when the number of animals is considered which, by actual experiment, he proved to be capable of producing sugar at the liver. They embrace the following classes: the mammals generally, the birds, a large proportion of both the osseous and the cartilaginous fishes, and some of the Gasteropods and acephalous Mollusks. And, a most positive proof, moreover, of its production in the organ in question, was seen in the fact that it was always detected in the liver of the human fœtus, after the fifth month; and farther still, that the fœtus, in Oviparia, which is entirely separated from the mother, afforded sugar from the liver, and from no other organ. In Herbivora, whose natural food contains a large proportion of saccharine and amylaceous material, the liver appeared not to furnish a very large amount

---

* The quantity of sugar formed at the liver, depends, beyond a certain amount, on the quantity of blood passing through it. More passing through during digestion, the production of sugar at that period is consequently increased.

of sugar, while the generation of fat by that viscus (another of Bernard's noble additions to the facts of science) was proportionally great. But in carnivora, which have a large supply of fat in their food, with but little or no sugar, it was evident that the sugar, found to exist in the liver, was generated *de novo.*

Bernard's first experiment, was made upon two dogs, one of which was killed seven hours after feeding heartily upon mutton and chicken-bones, and while digestion was going on actively. The blood from the right cavities of the heart, on standing for an hour and a half, yielded an opaline, milky serum, which, on the application of tests was found to contain sugar, while not the slightest evidence of its presence in the intestinal canal could be obtained, nor could it be detected in the urine. The other dog, full-grown and well-conditioned, was kept completely without nourishment for two days, and then killed. Again, the blood from the heart cavities afforded the saccharine serum, while the canal of the intestine, and the urine, were as free from it as before. It thus seemed evident that the blood contained sugar, independently of the nature of the ingesta, or of the change accomplished in digestion. The question then remaining to be determined, was the source, or organ, whence this sugar was derived, and for the solution of this problem, a second series of experiments was undertaken. A full-grown, healthy dog, was killed during active digestion, seven hours after feeding on mutton and chicken-bones. The abdomen being immediately opened, the digestive organs were seen to be turgid with blood, and the lacteals filled with chyle. The serum of blood, taken from the portal vein, near the entrance of the splenic vein, yielded a large quantity of sugar, on standing. Chyle from the thoracic duct, and chyme from the stomach and intestine, manifested no trace of it, while the serum of the blood from the cavities of the heart* readily manifested its presence, though not in so great abundance, as that from the V. portarum. Another dog was killed after a three days' fast. The organs of digestion were found pale and anæmic, but as in the other instance, though in somewhat less degree, sugar was found in the serum of the portal blood, as well as in that from the heart. Similar experiments, repeatedly made, confirmed these results; but from the great improbability that the walls of the V. porta. were the agents in the formation of the sugar, the question as to its origin still remained unsettled. Believing, however, that one of the great agents in effecting the portal circulation, was the compression exerted

* Of course here, as elsewhere, the right heart-cavities are meant.

by the abdominal walls, M. Bernard thought it not improbable, that when that compression was removed, a reflux of blood from the liver into the V. porta. occurred, whereby the constitution of the blood, normal to the vein, was changed. That his conjecture was a true one was readily established by the following experiment: A strong dog, while in full digestion of a meal of animal food, was killed by laceration of the medulla oblongata, the death, of course, being instant. The abdomen was quickly opened and ligatures immediately placed on the veins emanating from the small intestine. The vessels thus ligated were the splenic vein, the pancreatic veins, and the portal vein, just before its entrance to the liver. From all these localities, blood was obtained, and carefully tested, as also were the contents of the intestinal canal. No evidence of the presence of sugar was found in the blood from either of the veins, nor in the food from the digestive canal. But when an aperture was made in the portal vein, on the hepatic side of the ligature, the blood which regurgitated from the liver furnished the evidence of an abundant quantity of sugar. Farther still, on the application of reägents to the substance of the liver itself, it yielded abundant proof of saccharine formation, while examination of the spleen, pancreas, and mesenteric glands, afforded no such testimony. Hence it was very naturally and justly concluded, that the sugar found at the previous examinations in the V. porta. was due to regurgitation from the hepatic veins, in consequence of the withdrawal of the pressure of the abdominal walls. In order, therefore, to isolate the sugar as much as possible at the place of its production, it was obviously necessary to ligate the V. porta. close to the liver immediately on entering the abdominal cavity.* By this method of operating, sugar was detected, as before, when, for so long a time as six weeks, the animals had been entirely deprived of all saccharine or amylaceous ingesta. In a subsequent course of experiments, eight dogs were confined to different and exclusive articles of food. Upon four of them the experiment was continued for four days, and upon the other four for eight. "Of the four dogs in each series, one (A) was condemned to abstinence, as a point of comparison, B was nourished with fat only, having 125 grains daily; C had only gelatine, also 125 grains, and D was kept on fecula, 125 grains daily. Each had 300 grains of water daily. In the first series, killed at the expiration of four days, the following quantities of sugar were found in a hundred parts of

* This mode of procedure is essential to a successful performance of the operation. In one instance, where Dr. Dalton, designedly, delayed the application of the ligature, the sugar was found to extend a considerable distance from the liver blood, taken from the veins just after traversing the liver: A 0.96 grm'es, B 0.88 grm'es, C 1.65 grm'es, D 1.88 grm'es. In the second series, at the end of eight days continuance of the experiment, A gave 0.13, B 0.57, C 1.35, and D 1.50. It is evident from these cases, that the animals continued to produce sugar from gelatine and fecula, but that the fat did not serve for that purpose, the quantity of sugar produced being no greater in the dog nourished by fat than in the one condemned to abstinence."* We can say, then, that the sugar is formed at the expense of either azotized or saccharine matter. There is also some probability that it is the chief form in which the products of the disintegration of muscular and other albuminous tissues are made available for the maintenance of animal heat.

In order to the detection of sugar, in the hepatic tissue or in the blood, the following steps are necessary. If a portion of the fresh liver of an animal be beaten up in a mortar, and then boiled for a few moments in a small quantity of water, a sufficient quantity of carbonate of soda being added to prevent coagulation, the decoction will present an opaline appearance, and will exhibit all the characters of a saccharine solution. The serum of recent blood, from the right cavities of the heart, or from the hepatic veins, afford, when appropriately tested, as decisive proofs of the existence of sugar as the hepatic tissue itself. The presence of sugar in the fluid submitted to examination, may be detected by fermentation, by its action on silver or copper, reducing their oxides, by the formation of the saccharate of lime (Peligot's process) by means of polarized light (the method of Biot), but by far the most delicate and surest test, and the one generally employed by Bernard, is that of the double tartrate of potash and copper, known as "Barreswil's test-liquor." It is a modification of Trommer's test, the potash and copper being used together instead of separately. In making the test-liquor, in a pint of water is dissolved caustic potash, and chrystalized carbonate of soda, of each ℈iv., bi-tartrate of potash ℈v., sulphate of copper ℈iii. The solution is to be boiled, filtered, and kept in stoppered bottles. A few drops of this test, added to a strained decoction of a solid tissue, or to any other fluid, will, if grape sugar be present, on the application of a spirit lamp, throw down a reddish-yellow precipitate, the sub-oxide of copper. Of course the other sugars must be converted into grape sugar, in the application of this test. As the presence of any organic compound in the solution often interferes with the test, it will generally be advisable to add plumb. acet. q. s., and, after filtration (especially in experimenting on Herbivora) to add enough sulphuric

* Atlee.

acid to throw down any carbonates which may be present, then to filter again, and finish with the test-liquor.

All of Bernard's efforts to isolate this animal (or, as he calls it, diabetic) sugar, in a crystalline form have been unsuccessful. He attributes this to the presence of various salts, particularly to the chloride of sodium. Its chemical composition is identical with that of glucose, though it undergoes transformation much more readily. As it is unazotized, though it be derived from nitrogenous matter, the nitrogen must be separated in the liver, and carried off in the bile. Notwithstanding this relation, the variations in the quantity of the sugar, have no relation with those of the quantity of the bile. Though so nearly identical with glucose, this sugar, obtained from animal juices, manifests characters peculiar to itself, which are undoubtedly necessary to its special office in the economy. Bernard calls it diabetic sugar, from the analogy he finds to exist between it and the sugar yielded by the urine of those suffering that disease.

The supposition, objected by Dumas, to Bernard's theory, that the liver had the power of storing up within its tissue the products of previous sacchariferous ingesta, was shown to be incorrect, by depriving an animal entirely of food for eight days, then letting him have meat alone for twelve days, when he was killed. The sugar was found as abundantly as before in the hepatic tissue and right heart.

Bernard, also, discovered that by dividing the pneumogastrics or exhausting their energy, and by dividing the great sympathetic, the generation of sugar was immediately arrested. Indeed, all diseases, which exhaust nervous energy, have the same result, for which reason, it is never, or rarely found in the human liver, except in cases of sudden death. Even in the last stages of diabetes, during the exhaustion which precedes death, the sugar disappears from the urine. From the liver of a guillotined criminal, who had fasted for a day previous to his death, Bernard obtained ʒv. of sugar.

While pursuing these experiments, Bernard accidentally discovered that puncturation of the floor of the fourth ventricle, with a fine-pointed instrument, was followed, in a few minutes, by the appearance of sugar in the urine. The instrument was applied between the roots of the auditory and pneumogastric nerves, in the middle of the calamus scriptorius. Further research showed him that this was not the only part of the nervous centres, whose irritation produced an increase of the quantity of the sugar in the blood. The same effect followed, and even more strikingly, upon puncture of the corpora olivaria; indeed, with the dexterity consequent on frequent practice, he was able, with wonderful exactitude, to predict the degree of diabetes which would ensue according to the irritation applied to the nerve-centres. This fact, with many others observable in the phenomena of nervous irritation, prove the untenability of the theory of secretion put forth by Henle, "that secretions are all filtrations, depending upon the force of the blood and the resistance of tissues, the blood being contained in a vessel, and a wall existing between it and the cell-cavity into which the secretion is poured." If the irritation be made roughly, however, so as to involve any extensive lesion of the nervous structure, not only will the liver fail to augment the quantity of sugar in the blood, but for a time it will be deprived of the power of forming it. The *modus operandi* of this central irritation upon the liver is not yet conclusively established. The idea, at first entertained, that the stimulus conveyed by the pneumogastric to the hepatic tissue, excited it to a more energetic discharge of its functions, was overthrown by the fact that irritation of the corpora olivaria continued to produce its effects, after section of the pneumogastric was practiced. The present opinion is, that the great sympathetic is to be viewed as the means of the transmission of the effect of the stimulus; sufficient facts have not yet been observed, however, nor enough experimental results obtained, to establish this theory incontrovertibly.

When M. Bernard perceived that no sugar was to be detected in the current of blood flowing *into* the liver, while not only in the parenchyma of that organ, but in the hepatic veins, the ascending cava, and the right heart, its abundant presence was sufficiently manifest, while, again, in the pulmonary vein and left heart, if detected at all, its quantity was immensely diminished, it was very natural that he should conclude that it was fabricated in the liver and destroyed in the lungs. And that, as to the sugar itself, there were two separate and distinct sources whence it was obtained: the one, from ingesta, the other from the liver, as its proper and normal secretion. The sugar, then, being almost entirely lost after going into the lungs, what becomes of it, and how is it decomposed? Bernard's opinion is, that by a gradual fermentation, the sugar as it exists from the liver to the lungs, is converted into lactic acid ($C^6 H^5 O^5$), which, at the lungs, is changed into carbonic acid and water, by combustion, and exhaled. This special ferment has not, as yet, been isolated, but if it did not exist there, temperature would not exert the influence it does upon its properties. Its source is believed to be an organic principle, connected with the alkalinity of the blood.

It appears evident from the direct relation which exists between activity of respiration and the quantity of sugar formed at the liver, that this formation furnishes one of the conditions necessary to the proper performance of respiration. It was observed by M. Vernois (who submitted 173 livers to examination, for the purpose of verifying and extending Bernard's researches) that if artificial respiration be maintained in a decapitated animal, the production of sugar in the liver will still go on, while if the lungs be inflated with air mingled with chlorine or any other irritant vapor, the sugar appeared in the urine, the animal thus suffering diabetes after death. It is also an important and significant fact, that the blood, just before it is sent to the lungs for oxygenation, should be joined by a compound which, after oxygenation, exists in it no longer, or in a slight degree. It seems, therefore, reasonable to believe that the sugar is destroyed at the lungs, in order to minister to the function of respiration and to the maintenance of animal heat. Or, as Dr. Atlee concisely expresses it in his notes of M. Bernard's lectures: "When but little sugar is made, all is destroyed in passing the lungs. When more is formed, as during digestion it exists in the blood in an intermediate state, namely, lactic acid. And, when an excess is formed, it is not changed into lactic acid, but is carried into the arterial system, and thence into the venous. Hence if a man be bled while digestion is going on, sugar may be found in the blood taken from the veins of the arm, which will never be the case when he is fasting. Here is, then, an oscillation in the production of sugar, its occurrence in the arteries being exceptional. The blood must not contain more than one per cent. of sugar;* when it contains more than this amount, it is discharged by the urine. Of course all the intermediate products, between sugar and carbonic acid, may be found in the blood, constituting the variable principles existing in that fluid."

Remarking the general hypertrophied condition of the livers of diabetic subjects, and at the same time finding a very great excess over the normal amount, of sugar, in those livers,† Bernard inclined to look at the liver as the primary seat of the disease. In his opinion the excessive amount of sugar produced by the liver "exercises its metamorphic power upon the azötized constituents of the blood, thus destroying the material for the nutritive processes, an idea which corresponds well with the phenomena of the disease, which indicate an impoverishment of the nutritive fluids, while the solids exhibit a rapid waste, notwithstanding there may be an extraordinary appetite and a large amount of azotized nutriment taken in."‡

---

* That is, beyond the lungs.

† From one liver of a diabetic subject, he obtained the enormous amount of 833 grains of sugar.

‡ Carpenter.

